# Microbial community assembly and functional potential response driven by soil phosphorus gradients

**DOI:** 10.3389/fmicb.2026.1818929

**Published:** 2026-06-26

**Authors:** Shuang Peng, Beibei Zhou, Xiangui Lin, Jinping Guo, Yiming Wang

**Affiliations:** 1 College of Environment and Ecology, Jiangsu Open University, Nanjing, Jiangsu, China; 2 State Key Laboratory of Soil and Sustainable Agriculture, Institute of Soil Science, Chinese Academy of Sciences, Nanjing, Jiangsu, China; 3 Jiangsu Collaborative Innovation Center for Solid Organic Waste Resource Utilization, Nanjing, Jiangsu, China; 4 Fujian Tobacco Research Institute, Fuzhou, Fujian, China

**Keywords:** acidic soil, bacterial community, phosphatase activity, phosphorus cycling, tobacco-growing soil

## Abstract

Understanding how long-term phosphorus (P) fertilization reshapes soil microbiomes and their functional interplay with P cycling is crucial for sustainable agriculture. This study investigated the effects of different P levels on soil physicochemical properties, phosphatase activities, and the bacterial community across three geographically distinct tobacco-growing sites (YD, SH, and ZS) in Fujian Province, China. Our results revealed a significant positive correlation between soil available phosphorus (AP) and phytase (*R* = 0.92), challenging the classical P deficiency induction paradigm. Site-specific environment was the paramount factor overriding contemporary P levels in shaping the bacterial community structure and its P-cycling genetic potential. At the broadest scale, stochastic processes dominated the assembly of regional species pools, leading to distinct community templates across sites. Within each site, however, the local P levels acted as a deterministic filter, fine-tuning community composition primarily through species replacement rather than wholesale restructuring. Functional prediction revealed site-specific P-cycling strategies: the YD community showed a higher predicted genetic potential toward aggressive P acquisition (mineralization and solubilization), whereas SH and ZS communities exhibited a higher predicted potential for P scavenging, storage, and recycling (polyphosphate degradation and transport). Redundancy analysis linked these functional profiles to distinct local soil properties: organic matter (OM) at YD, a combination of OM and multiple nutrients at SH, and potassium at ZS. In conclusion, microbial-mediated P cycling in these soils may be governed by a hierarchical mechanism: site-specific context sets the community template, localized soil properties (OM, K) shape the functional repertoire, and current P management modulates the system through species sorting and likely transcriptional regulation. This underscores the need for site-specific, ecological precision management strategies that target dominant local environmental drivers to foster microbial communities capable of optimizing soil P efficiency.

## 1 Introduction

Phosphorus (P) is a critical macronutrient essential for plant growth and agricultural productivity (Wyngaard et al., 2016). However, its low availability in many soils, especially acidic soils, often limits crop yield (Gerke, 2015; Lambers, 2022). To overcome this limitation, mineral P fertilizer application has become a common practice worldwide, which has led to significant P accumulation in soils (Chowdhury et al., 2017) and caused associated environmental concerns, such as water eutrophication (Hu et al., 2018; Yuan et al., 2025). Consequently, a paradox has emerged in some intensively managed agricultural systems: soils become rich in total P yet remain deficient in plant-available P, a situation that can trigger a cycle of continued fertilizer application. The tobacco-production soils of Fujian Province, China, serve as a typical example of this paradox, where high legacy P coexists with plant P limitation. Without additional P fertilizer, tobacco plants often exhibit stunted growth after transplantation (Zhou et al., 2024). Therefore, understanding the mechanisms behind this paradox, particularly the role of soil microbial communities in regulating P bioavailability under long-term fertilization, is crucial for developing sustainable P management strategies.

In soil ecosystems, the transformation and availability of P are profoundly mediated by microorganisms (Park et al., 2022). Bacteria and archaea drive the biogeochemical P cycle through a suite of processes, including the solubilization of mineral P, mineralization of organic P, intracellular storage as polyphosphate, and regulatory responses to P stress (Richardson and Simpson, 2011; Bergkemper et al., 2016). Key enzymes such as phosphatases (e.g., phytase, alkaline/acid phosphatases) hydrolyze organic P compounds, making inorganic phosphate available for uptake by plants and microbes (Margalef et al., 2021). The genetic potential for these functions is encoded in diverse microbial taxa, and their expression shapes the soil's capacity to supply plant-available P (Xing et al., 2021; De Almeida Leite et al., 2024).

A classic ecological principle holds that microbial phosphatase activity is typically upregulated under low-P conditions as a stress response to scavenge P, leading to a negative correlation with soil available P (Ragot et al., 2017; Samaddar et al., 2019). However, this paradigm is increasingly challenged in managed agricultural ecosystems. Long-term fertilization can alter soil physicochemical properties, such as pH, organic matter (OM) content, and cation balances, creating novel selective environments that reshape microbial community structure and function (Geisseler and Scow, 2014; Hartmann and Six, 2023; Zhang et al., 2024). Emerging evidence suggests that in such human-modified systems, the relationship between P availability and microbial function can be complex and even paradoxical, potentially shifting from deficiency-driven to substrate-driven strategies (Dai et al., 2020; Zhang et al., 2023). Understanding these context-dependent responses is crucial for predicting soil P dynamics under continuous management.

The assembly of soil microbial communities, which underpin P transformation functions, is governed by both deterministic (e.g., environmental filtering, biotic interactions) and stochastic processes (e.g., dispersal, drift; Dini-Andreote et al., 2015; Stegen et al., 2012). A key question is how geographic location, with its inherent legacy of soil type, parent material, and management history, interacts with contemporary P management to filter the regional species pool and guide community assembly. The concept of “everything is everywhere, but the environment selects” underscores the power of local conditions (de Wit and Bouvier, 2006). Addressing the paradox of high total P but low bioavailability in Fujian's tobacco soils requires a systemic understanding of the soil microbiome's role. Previous studies on P cycling have often focused on single locations, specific microbial groups, or isolated processes. An integrated assessment linking in-field P gradients across different geographic settings to changes in soil chemistry, microbial community structure, assembly mechanisms, functional gene potential, and extracellular enzyme activity is critically lacking for this economically important system.

Therefore, based on a pre-survey, this study selected farmland soils with varying historical P fertilization levels across three representative tobacco-growing regions in Fujian: Yongding (YD), Shanghang (SH), and Zhongsha (ZS). We aimed to perform a detailed comparative analysis to identify key ecological associations across sites that may reflect the long-term effects of differential P fertilization level. The specific objectives were to: (i) characterize the variations in soil physicochemical properties and phosphatase activities across the P gradient at each site; (ii) analyze the shifts in bacterial community diversity, composition, and co-occurrence patterns in response to different soil P levels; (iii) predict the changes in genetic potential for key P-cycling functional processes within the bacterial communities; (iv) decipher the dominant processes (stochastic vs. deterministic) governing bacterial community assembly under different P levels; and (v) identify the key soil physicochemical factors driving the observed changes in community structure and function. This study seeks to provide a mechanistic, microbiome-centric perspective on P cycling in high-P tobacco soils, offering a scientific basis for developing precise P management strategies that enhance P-use efficiency and promote soil health in Fujian's tobacco-growing system.

## 2 Materials and methods

### 2.1 Site descriptions, soil collection and preparation

Sampling was conducted in three long-term tobacco-growing regions of Fujian Province: Baidong Village (Hulei Town, Yongding District), Fengji Village (Lufeng Township, Shanghang County), and Xiasha Village (Zhongsha Township, Ninghua County). Prior to this study, a broad survey of soil available-P was performed across these regions. Two separate zones with contrasting soil available-P levels (one relatively high and one relatively low) were selected within each region. This resulted in a total of six study zones. The background available-P levels and crop-rotation patterns for these six pre-selected zones are summarized in Table 1. The classification of soil P levels (low, medium, and high) was defined relative to the local P gradient at each site, reflecting differences in baseline soil P status. Therefore, statistical comparisons and ecological inferences are primarily drawn from within-site contrasts between relative P levels.

**Table 1:** Background characteristics of the six pre-selected tobacco sites, tobacco varieties, and cropping patterns across the three study regions.

Site	P Level	Total P (g/kg)	Available P (mg/kg)	Variety	Soil type	Cropping pattern
Baidong Village, Hulei Town, Yongding (YD)	Medium (M)	0.63 ± 0.09	37.99 ± 6.86	Yunyan 87	Sandy Loam	Previous rice
	High (H)	0.84 ± 0.07	74.69 ± 0.90	Yunyan 87	Sandy Loam	Previous rice
Fengji Village, Lufeng Town, Shanghang (SH)	Medium (M)	0.67 ± 0.06	44.75 ± 2.64	Yunyan 87	Loam	Previous rice
	High (H)	0.79 ± 0.01	70.95 ± 8.41	Yunyan 87	Loam	Previous rice
Xiasha Village, Zhongsha Town, Ninghua (ZS)	Medium (M)	0.8 ± 0.10	27.67 ± 2.90	CB-1	Loam	Previous rice
	Low (L)	0.69 ± 0.04	22.99 ± 1.74	CB-1	Loam	Previous rice

Data represent mean (± SD) values from six sites pre-selected within each region (three high-P and three low-P sites) prior to the intensive sampling.

During the vigorous-growth stage of tobacco, five representative plots were selected within each P-level zone at each site. Within each plot, three spatially separated sampling points were established. At each point, five soil cores (0–20 cm depth) were collected near the root zone (approximately 5 cm away from the plant stem) using a core sampler (20 mm inner diameter), and thoroughly homogenized to form one composite subsample. Thus, three independent composite subsamples were obtained from each plot, preserving within-plot spatial variability. This design resulted in a total of 15 samples per “site × P-level” combination for downstream analysis. The collected soil was thoroughly homogenized after the roots and gravel were removed. Approximately 500 g of soil was divided into two parts. The first part was stored in a refrigerator at low temperature and quickly transported to the laboratory, where it was stored at −40°C until it was used for the extraction of total DNA from the soil. The second part was air-dried at 22–30 °C and passed through meshes of 2 and 0.149 mm for the determination of soil properties.

### 2.2 Soil phosphatase activities determination

The soil phytase, soil acid phosphatase (AcP), and soil alkaline phosphatase (AlP) activities were determined using Solarbio soil phytase kit (Solarbio, BC5375), soil acid phosphatase kit (Solarbio, BC0145), and soil alkaline phosphatase kit (Solarbio, BC0285). Based on the provided kit instructions, the activity of soil phosphatases was determined as follows and expressed in units per gram of dry soil (U/g).

AcP and AlP activities were measured using the phenyl disodium phosphate method. Briefly, 0.1 g of air-dried soil was incubated with 0.4 mL of the corresponding pH-specific buffer and 0.05 mL of toluene for 24 h at 37 °C. The released phenol was colorimetrically quantified at 660 nm after reaction with 2,6-dibromobenzoquinone chlorimide under alkaline conditions. Enzyme activity, based on a phenol standard curve, is defined as the amount of enzyme that releases 1 nmol of phenol per day at 37 °C. Phytase activity was determined by quantifying inorganic P released from sodium phytate. A total of 0.03 g of air-dried soil was incubated with 20 μL toluene and 500 μL substrate solution for 24 h at 37 °C. The reaction was terminated by boiling for 10 min. The generated inorganic P reacted with a molybdenum blue working solution, and the absorbance was measured at 700 nm. Activity was calculated from an inorganic P standard curve and is defined as the amount of enzyme that releases 1 μmol of inorganic P per day at 37 °C.

### 2.3 Soil chemical characteristics analysis

Total nitrogen (TN) was determined using the modified Kjeldahl method. Total phosphorus (TP) was extracted via H_2_SO_4_-HClO_4_ digestion and subsequently quantified by spectrophotometry. Total potassium (TK) was measured using the NaOH fusion–flame photometer method. Dissolved total nitrogen (AN) in soil was extracted with 2 M KCl and analyzed using a continuous flow analytical system (San++ System, Skalar, Holland). Available phosphorus (AP) was extracted with sodium bicarbonate and measured by the molybdoantimonyl colorimetric method (Olsen method). Available potassium (AK) was extracted with 1.0 M ammonium acetate (pH 7.0; 1:10 soil: solution, 30 min shaking), slow-release potassium (SRK) was extracted by 1.0 M HNO_3_ hot extraction (1:10 ratio, 90 °C, 1h). Extracts were filtered through 0.45 μm membranes and analyzed using a flame photometer. Organic matter (OM) was analyzed using the potassium dichromate volumetric method. Soil pH was measured with a glass combination electrode pH meter (REX, Shanghai, China) at a water-to-soil ratio of 2.5:1.

### 2.4 Soil DNA extraction and Illumina MiSeq DNA sequencing

Total genomic DNA of the microbial community was extracted using the FastDNA^®^ SPIN Kit (MP Biomedicals), following manufacturer protocols. DNA concentration and purity were measured with an ND-1000 spectrophotometer (Thermo-Scientific), and stored at −20 °C.

The microbial communities in soil samples were analyzed using high-throughput sequencing method conducted on an Illumina NovaSeq6000 platform (Novegene, Beijing, China) using a 2 × 250 bp paired-end strategy. The V4–V5 region of the bacterial 16S rRNA gene was amplified with universal primers 515F (5′-GTGCCAGCMGCCGCGGTAA-3′) and 907R (5′-CCGTCAATTCCTTTGAGTTT-3′). Sequencing libraries were generated using NEB Next^®^ Ultra™ II FS DNA PCR- free Library Prep Kit (New England Biolabs, USA) following manufacturer's recommendations and indexes were added. Effective Tags were obtained after paired-end reads assembly and quality control, then denoise was performed with DADA2 in the QIIME2 software (Version QIIME2-202006) to obtain initial ASVs (Amplicon Sequence Variants). The average final read length after processing was 373 bp. An average of 105,338 high-quality reads per sample were retained for downstream analysis. Species annotation was performed using QIIME2 software. All ASVs (including 2% archaeal sequences) were retained for the following analysis. Alpha diversity indices, including Shannon, Simpson, Chao1, and ACE, were calculated with QIIME2. The sequences were submitted to the NCBI Sequence Read Archive (SRA) database (accession no.: PRJNA1460904).

### 2.5 Statistical analysis

Alpha diversity was calculated based on the rarefied ASVs table using the vegan package. The Bray-Curtis distance matrix was calculated and the β-diversity of the community was analyzed by Principal coordinate analysis (PCoA). Permutational multivariate analyses of variance (PERMANOVA) were used to examine the difference in community structure. Differential abundance analysis of ASVs between soils with different P levels was performed using the R package DESeq2. Prior to analysis, the ASV count table was pre-filtered to retain features with a total count ≥20 across all samples and presence in at least 20% of the samples within any comparison group. ASVs with a false discovery rate (FDR) adjusted *p*-value < 0.05 and an absolute log2 (fold change) >2 were considered significantly differentially abundant. To explore the relationships between the enriched ASVs and environmental conditions, Spearman correlation analysis was conducted between the relative abundance of these ASVs and environmental variables at each site. The resulting *p*-values from all pairwise correlations were adjusted for multiple testing using the Benjamini-Hochberg procedure to control the FDR. Correlations with an FDR-adjusted *p*-value (*p*) < 0.05 were considered statistically significant. Functional prediction of the bacterial community was performed using the PICRUSt2 (V2.3.0) software (Langille et al., 2013), and P cycling functional genes were screened based on the list provided in Supplementary Table S1. Genes involved in soil microbial P-transformation in different treatments were searched for in the datasets based on previous publications (Chen et al., 2025). Pre-filtered ASVs with strong and significant relationships (correlation coefficient |r| > 0.70 and *p* < 0.05) were constructed in networks based on Spearman correlation test using the “Hmisc” package. The neutral community model (NCM) was utilized to evaluate the potential influence of stochastic processes on the assembly of bacterial communities with “MicEco” package (Cheng et al., 2024). All analyses were conducted using R version 4.3.2, employing the “linkET” package for Mantel tests, the “vegan” package for redundancy analysis (RDA), PCoA, and the “pheatmap” package for spearman correlation analysis.

## 3 Results

### 3.1 Variations in soil physiochemical properties and soil phosphatases

**Figure 1:**
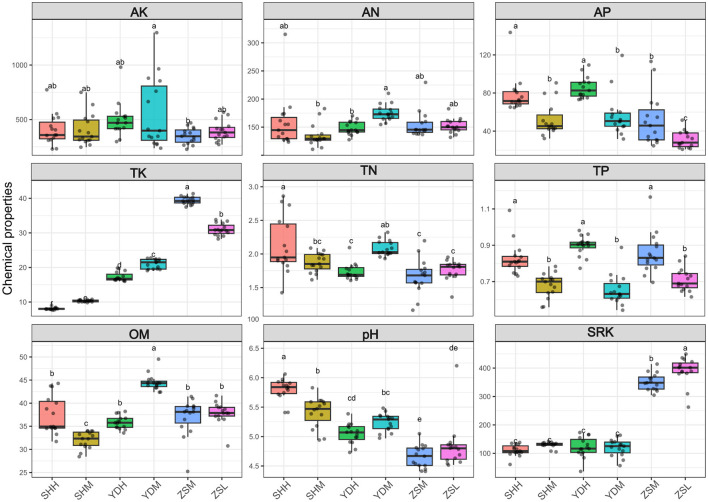
Soil physiochemical properties. Different letters indicate significant differences based on one-way ANOVA followed by a Fisher test (*n* = 15 per group). YD, Yongding; SH, Shanghang; ZS, Zhongsha. The letters H, M, and L following the site abbreviations denote high, middle, and low levels of soil available phosphorus (AP), respectively.

Soil chemical properties are shown in Figure 1. The contents of AP and TP differed significantly among the three sites under different P levels, and their concentrations were generally consistent with the classified soil P levels. In contrast, the contents of AN and AK showed relatively small variations across regions and P levels. Furthermore, significant differences were observed in TK, TN, OM, and pH across sampling sites and P levels. Interestingly, the trends of change for OM, TN, and pH exhibited distinct regional patterns: they aligned positively with P levels at the SH site, showed an inverse relationship at the YD site, and displayed no significant variation across P levels at the ZS site (Figure 1).

**Figure 2:**
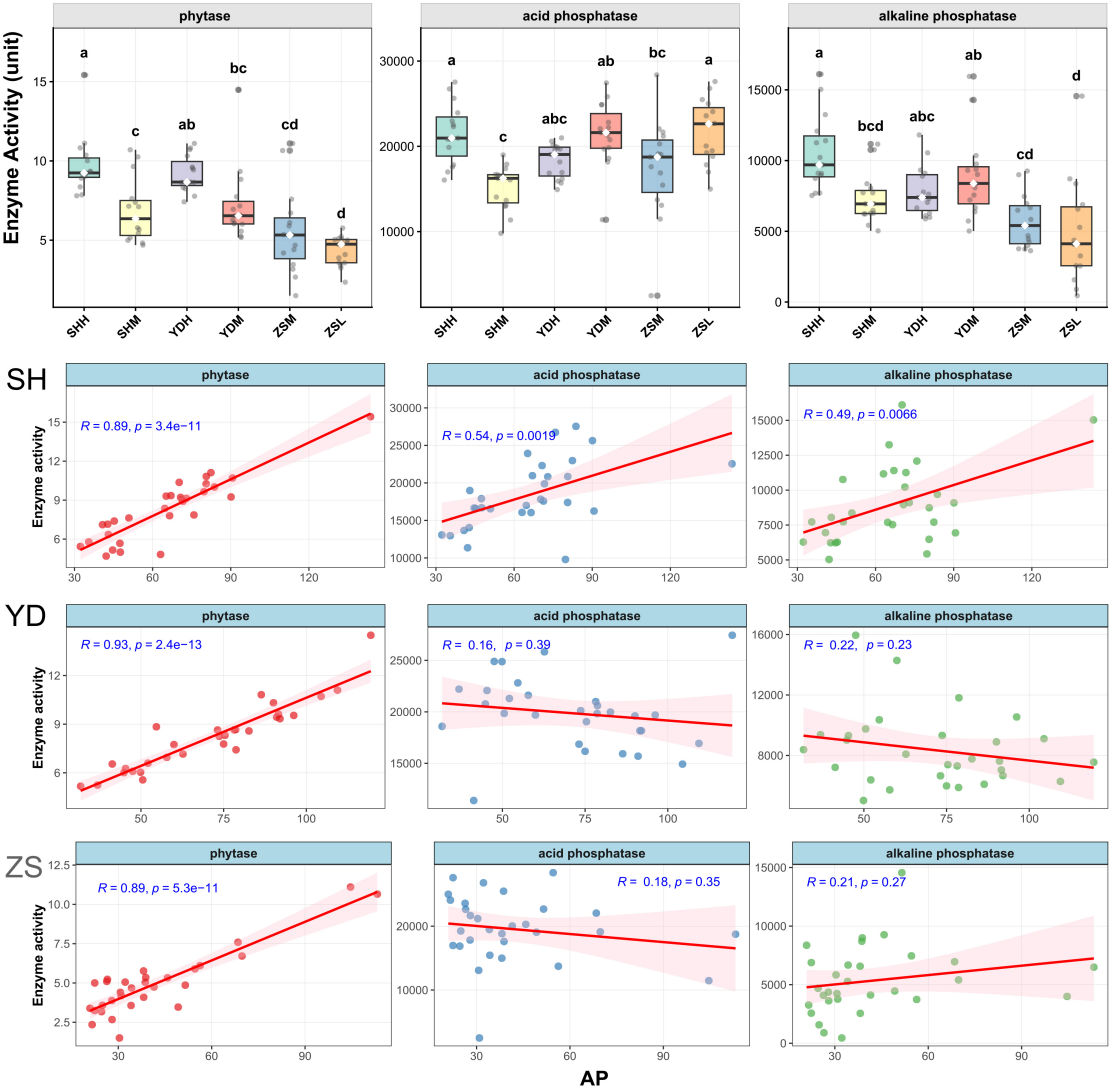
Soil phosphatases. Activity of three phosphatases and their correlation with soil available phosphorus (AP) under different phosphorus levels across three sites (*n* = 15 per group). YD, Yongding; SH, Shanghang; ZS, Zhongsha. The letters H, M, and L following the site abbreviations denote high, middle, and low levels of soil available phosphorus (AP), respectively.

The activities of three types of phosphatases in soils with different P levels across three sites were shown in Figure 2. Significant variations were revealed in phytase activity among different regions and P levels. Notably, phytase activities showed clear and consistent significant positive correlations with soil AP content across three sites (Figure 2), while the relationships for acid and alkaline phosphatases are either weak or context-dependent.

### 3.2 Variations in bacterial community diversity and composition

**Figure 3:**
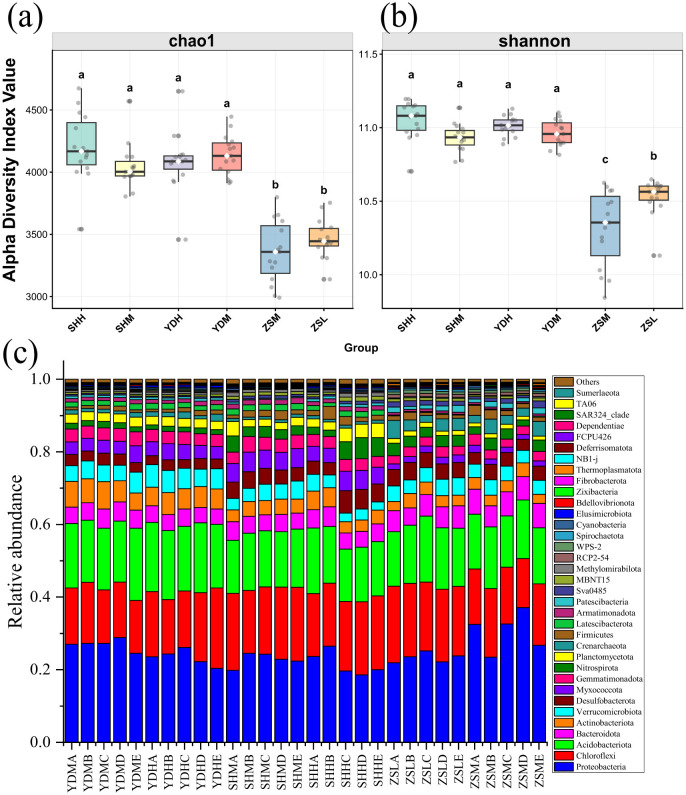
The diversity and composition of the bacterial communities. Bacterial community diversity indices **(a, b)** and composition in soils with different phosphorus levels across three sites **(c)**. Different lowercase letters after data represent significant differences at the *p* < 0.05 level, as tested by Tukey's test (*n* = 15 per group). YD, Yongding; SH, Shanghang; ZS, Zhongsha. The letters H, M, and L following the site abbreviations denote high, middle, and low levels of soil available phosphorus (AP), respectively. The letters A~E represent sampling points under different phosphorus levels across three sites (*n* = 3 per point).

Alpha diversity across bacterial communities were shown in Figure 3. Soils from ZS group (ZSL, ZSM) generally showed lower bacterial diversity (Shannon index: 9.8–10.65) and richness (Chao1: 2991–3798) compared to soils from YD and SH. Bacterial communities were predominantly composed of Proteobacteria (18.57%−37.11%), Chloroflexi (13.46%−22.15%), Acidobacteriota (14.12%−19.89%), Bacteroidota (4.21%−6.92%), Actinobacteriota (2.5%−7.53%), and Verrucomicrobiota (2.3%−6.24%; Figure 3). The relative abundances of these main bacterial phylum were not significantly different among the six groups (Supplementary Figure S1).

**Figure 4:**
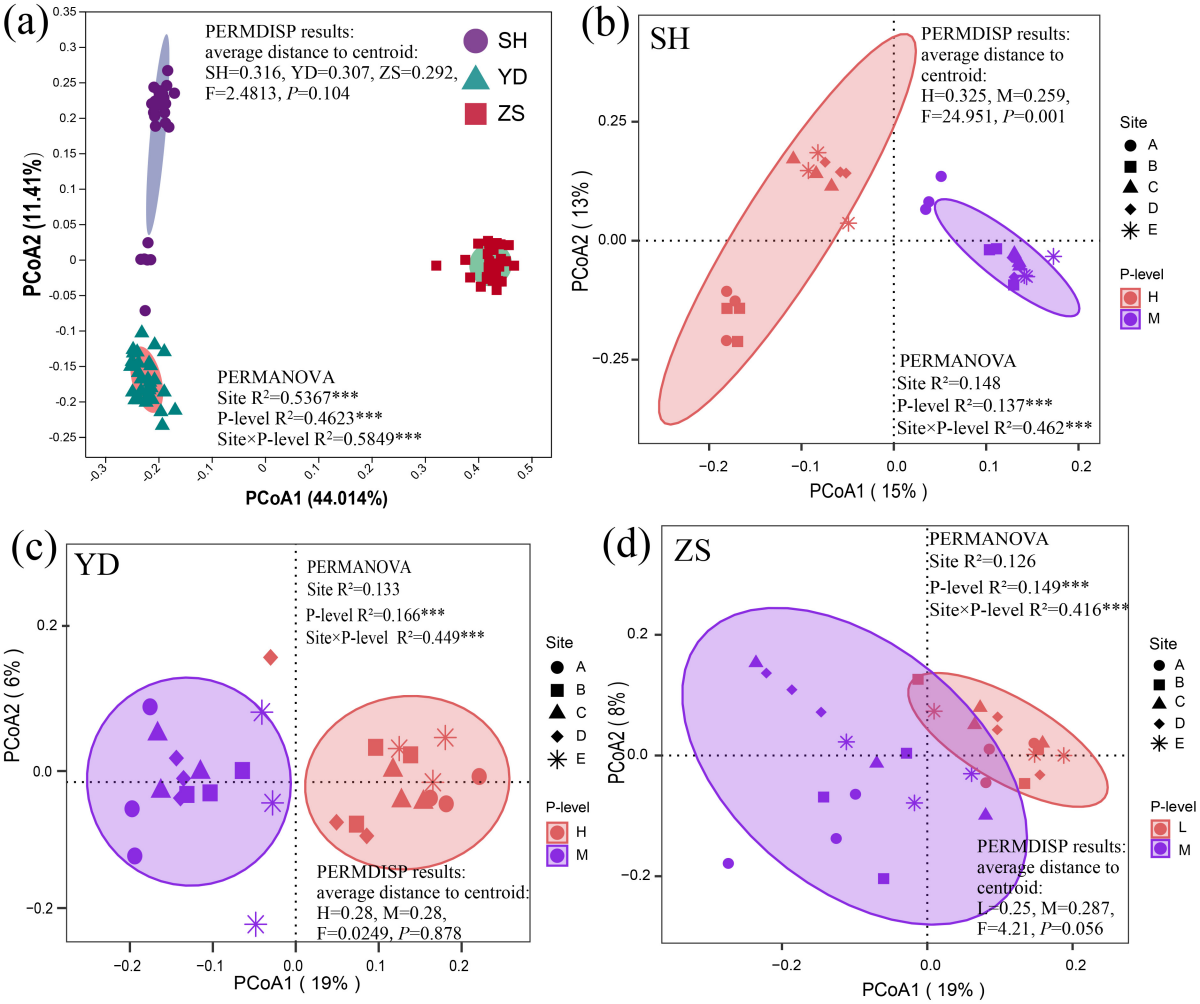
PCoA of bacterial community. Principal coordinates analysis (PCoA) of the species composition of bacterial communities in all soils **(a)** and soils from SH **(b)**, YD **(c)** and ZS **(d)**. YD, Yongding; SH, Shanghang; ZS, Zhongsha. The letters H, M, and L denote high, middle, and low levels of soil available phosphorus (AP), respectively.

**Figure 5:**
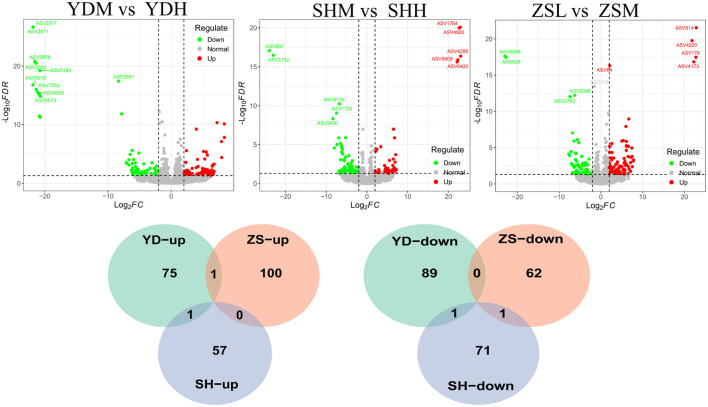
Volcano maps. Volcano plots (a,b,c) showing the significantly enriched or depleted ASVs between soils with higher available phosphorus and soils with lower available phosphorusat the same site. Red dots indicate significantly upregulated ASVs, green dots indicate significantly downregulated ASVs, and gray dots represent ASVs with no significant difference (DESeq2, *p* < 0.05, FDR adjustment). FC, fold change. YD, Yongding; SH, Shanghang; ZS, Zhongsha. The letters H, M, and L following the site abbreviations denote high, middle, and low levels of soil available phosphorus (AP), respectively (*n* = 15 per group).

PCoA of the bacterial community were shown in Figure 4, the results indicate significant differences in soil bacterial communities among the three sites. Furthermore, significant differences were also observed in bacterial communities among soils with different P levels at the same site. Detailed description of the PERMDISP results were explicitly linked to the PCoA (Figure 4). For the SH site, we clarify that the significant PERMANOVA result between different P level (*R*^2^ = 0.137, *p* < 0.001) reflects both differences in community centroids and heterogeneous dispersion. For YD and ZS, we note that the PERMANOVA significance is primarily driven by centroid differences, as dispersion homogeneity was not violated (or showed only a weak trend). Consequently, we compared soils with higher and lower P levels within each site. As shown in Figure 5, 100 ASVs were enriched in soils with higher P at the ZS site, while 57 and 75 ASVs were enriched at the SH and YD sites, respectively. Only a few enriched bacterial ASVs were shared across the three sites, indicating that the enriched ASVs exhibited distinct location-specific characteristics.

To further understand the potential contribution of bacteria enriched in soils with higher available P content to soil P levels, we analyzed the relationships between the relative abundance of enriched ASVs and soil chemical properties as well as phosphatase activities in each site (Supplementary Figure S2). Some ASVs exhibited significant correlations with AP and phytase activity, suggesting that these bacterial ASVs may play an important role in soil P activation processes. At the ZS site, ASVs showing significant positive correlations with AP were primarily derived from Proteobacteria (13/28). The ASVs exhibiting higher correlations with AP were identified as Pseudomonas genera (ASV2822, Proteobacteria, *r* = 0.558, *p* = 0.0014), Diplorickettsiaceae family (ASV2414, Proteobacteria, *r* = 0.652, *p* < 0.001), and Armatimonadota phylum (ASV7347, *r* = 0.555, *p* = 0.0014). At the SH site, ASVs exhibiting significant positive correlations with AP were primarily affiliated with Chloroflexi (4/17). The ASVs showing higher correlations with AP were identified as Gemmatimonadaceae family (ASV402, Gemmatimonadota, *r* = 0.557, *p* = 0.0014), Nostocaceae family (ASV5694, Cyanobacteria, *r* = 0.526, *p* = 0.0028), and Gaiellales order (ASV3667, Actinobacteriota, *r* = 0.516, *p* = 0.0035). At the YD site, ASVs exhibiting significant positive correlations with AP were primarily affiliated with Chloroflexi (5/13). The ASVs showing higher correlations with AP were identified as Anaerolineaceae family (ASV2532, Chloroflexi, *r* = 0.709, *p* < 0.001), Candidatus_Solibacter genera (ASV5834, Acidobacteriota, *r* = 0.638, *p* < 0.001), and Ktedonobacteraceae family (ASV3467, Chloroflexi, *r* = 0.599, *p* < 0.001). One archaeal ASV assigned to Bathyarchaeia genera (ASV2209, Crenarchaeota, *r* = 0.601, *p* < 0.001) was also highly correlated with AP in YD site.

### 3.3 The predicted functions of the bacterial community involved in P cycling

**Figure 6:**
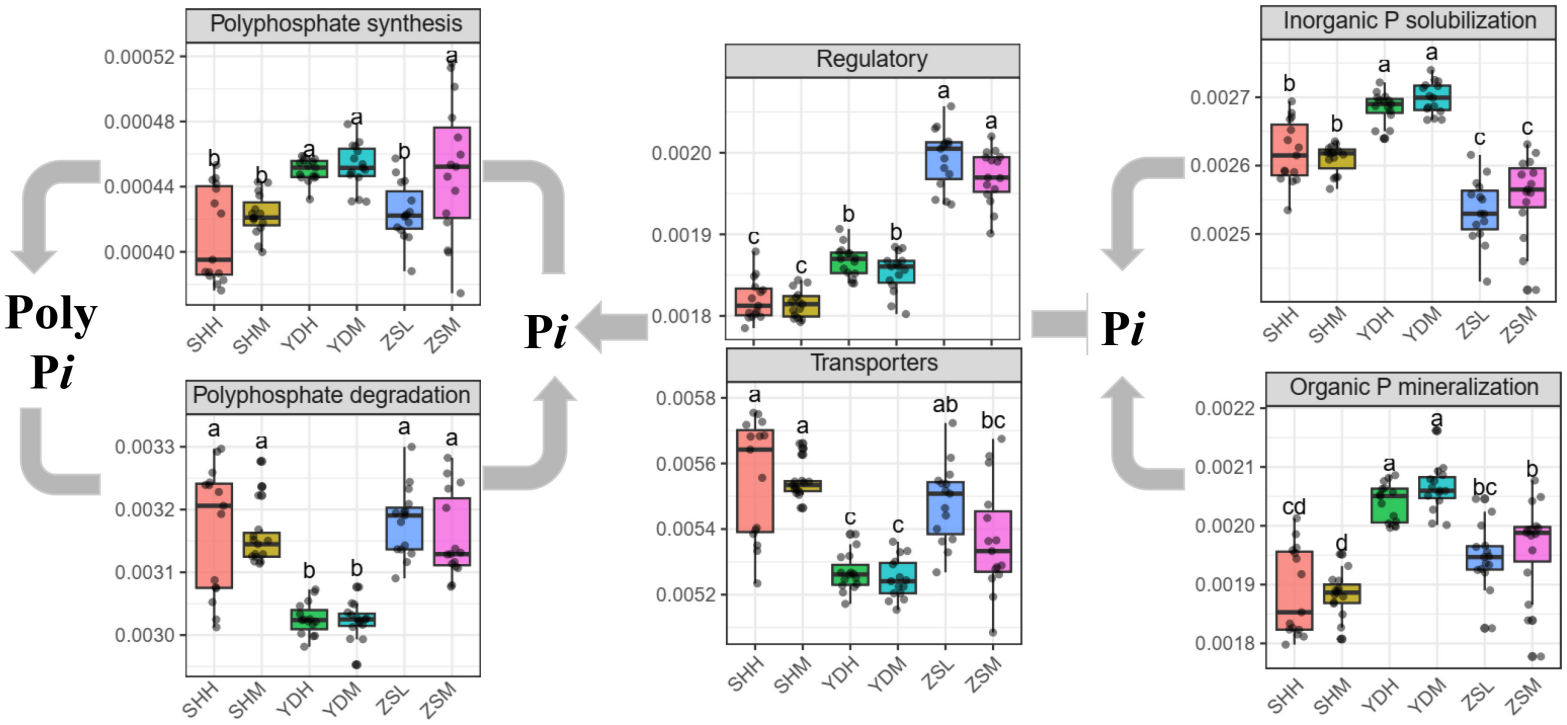
Predicted functions of the bacterial community involved in P cycling. Microbial metabolic pathways involved in P cycling. Boxplots show the total relative abundance of functional genes in different soil groups, based on functional prediction data of the bacterial community using the PICRUSt2. YD, Yongding; SH, Shanghang; ZS, Zhongsha. The letters H, M, and L following the site abbreviations denote high, middle, and low levels of soil available phosphorus (AP), respectively.

The total abundance of six categories of P metabolism functional genes is presented in Figure 6. The results *via* PICRUSt2 showed that the functional potentials for organic P mineralization, inorganic P solubilization, and polyphosphate synthesis significantly higher at the YD site compared to the other two sites. In contrast, polyphosphate degradation and transporters functional potentials were significantly higher at the SH and ZS sites than at the YD site. Additionally, the regulatory function was significantly higher at the ZS site than at the YD and SH sites. These results *via* PICRUSt2 suggest that P metabolism follows distinct pathways at different sites. The microbial community at the YD site harbors a stronger genetic potential for integrated P acquisition (i.e., genes related to both organic P mineralization and inorganic P solubilization), which may represent a key microbiological trait for maintaining soil P availability at this site, whereas the ZS and SH sites may rely more on polyphosphate degradation. The higher regulatory function at the ZS site may be attributed to its lower overall P content compared to the other two sites.

### 3.4 The assembly mechanism of bacterial communities under different levels of phosphorus

**Figure 7:**
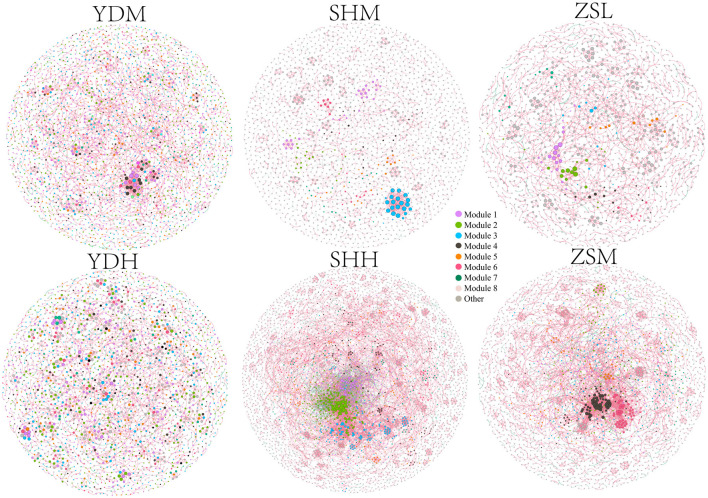
Network of the bacterial communities in soils with different phosphorus levels across three sites (*n* = 15 per group). Positive and negative correlations between nodes are indicated by red and green lines. The size of network nodes represents the node degree, and nodes in network are colored by modularity. YD, Yongding; SH, Shanghang; ZS, Zhongsha. The letters H, M, and L following the site abbreviations denote high, middle, and low levels of soil AP, respectively.

We constructed network graphs for bacterial communities in soils with different P contents collected from different regions. The results are shown in Figure 7. Different P levels had no significant impact on the bacterial network at the YD site. However, in soils with higher AP at the SH and ZS sites, the networks exhibited a significantly greater number of edges (i.e., higher density) compared to their lower-AP counterparts (Figure 7, Supplementary Table S2). Concurrently, the average clustering coefficient, which quantifies the tendency of nodes to form tightly interconnected triads, was significantly lower in these high-AP soils.

Using the neutral community model (NCM), we analyzed the assembly processes of bacterial communities in six groups of soil samples (Supplementary Figures S3a, S4). The results indicated that the bacterial communities in all six soil groups closely followed the neutral model, suggesting that the assembly of bacterial communities was largely influenced by stochastic processes. β-diversity partitioning analysis revealed that soil bacterial communities were positioned near the replacement or similarity vertexes (Supplementary Figure S3b), indicating that community differences were primarily driven by species replacement. When the six soil groups were analyzed separately, similar results were obtained (Supplementary Figure S5). This suggests that the influence of different P levels on the assembly of soil bacterial communities is mainly governed by stochastic processes, with changes in P content leading to the replacement of certain species.

### 3.5 The effect of soil physicochemical properties on the bacterial communities

**Figure 8:**
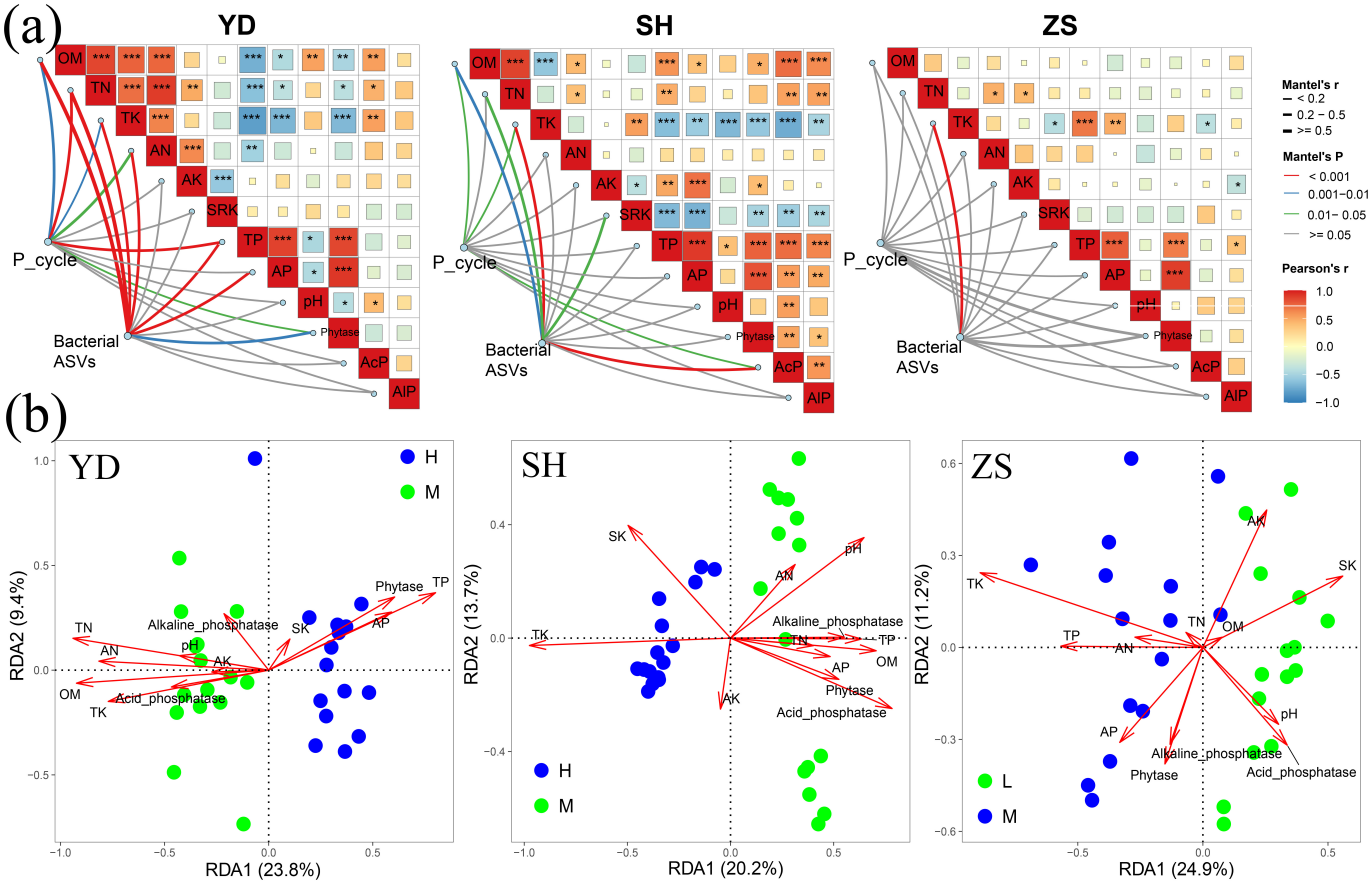
Mantel test and RDA. **(a)** Mantel test results showing correlations between environmental factors and P cycle function and bacterial ASVs profiles in soils with different phosphorus levels across three sites. Line width represents the Mantel's *r* statistic, while line color denotes statistical significance. **(b)** RDA scatter plots of the bacterial communities and the properties in soils with different phosphorus levels across three sites. Abbreviations for soil physico-chemical properties are same with Figure 1.

We analyzed the main factors influencing bacterial community structure and P cycling functional genes under different P levels at the three sites. Mantel test results indicated that at the YD site, soil bacterial community structure showed highly significant positive correlations with the contents of TP, AP, AN, TK, TN, and OM (*p* < 0.01). At the SH site, the main factors correlated with the bacterial community were TK, AcP, OM, TN, and SRK, while at the ZS site, only TK exhibited a highly significant positive correlation with the bacterial community. Among these environmental factors, some showed significant positive correlations with P cycling genes, such as OM and TK at the YD and SH sites (Figure 8a).

To further elucidate the relative importance of each environmental factor, RDA was performed (Figure 8b). These environmental factors collectively explained 46.25, 47.62, and 47.61% of the bacterial community variation at the YD, SH, and ZS sites, respectively. OM was the most significant factor explaining community variation at the YD site (*p* < 0.001). For the SH site, OM (*p* < 0.001), TK (*p* < 0.01), TN, AK, and TP (*p* < 0.05) were the main driving factors, whereas the key drivers at the ZS site were TK (*p* < 0.001) and AK (*p* < 0.05).

The Mantel test and RDA results revealed the influence of environmental factors on bacterial communities from different perspectives. The Mantel test indicated that at the YD site, the combined fertility gradient formed by soil TP, AP, AN, TK, TN, and OM content exhibited a highly significant synergistic association with changes in bacterial community structure. However, RDA further parsed the independent contributions of each factor and identified OM as the most significant driver of community variation. This apparent inconsistency actually reflects the intrinsic coupling of soil nutrients in agricultural ecosystems. At the YD site, OM likely serves as the core foundation of the soil habitat, with its content strongly covarying with other nutrients such as nitrogen and P due to long-term fertilization practices. While multiple factors collectively shape the microbial environment (as reflected by the Mantel test), OM represents the most informative and dominant variable underlying this co-variation pattern (as highlighted by RDA). Furthermore, the distinct key drivers identified across sites: OM at YD, a combination of OM and multiple nutrients at SH, and TK and AK at ZS, underscore the strong regional specificity of environmental filtering. This aligns with the concept that local soil legacy (e.g., parent material, historical management) creates unique ecological filters, selecting for microbial communities adapted to locally dominant resource conditions. The significant correlation between certain factors (e.g., OM, TK) and P cycling genes further suggests that these key environmental drivers may not only influence general community structure but also modulate specific functional traits related to P transformation, potentially creating a feedback loop between soil chemistry, microbial composition, and biogeochemical function.

## 4 Discussion

### 4.1 Chemical and phosphatases responses under soil phosphorus gradient

This study found that the contents of soil AP and TP differed significantly across the pre-established P level gradient, confirming the validity of the field experimental design. More notably, we observed significant differences across the P gradient not only in AP and TP, but also in other fundamental soil chemical properties (e.g., OM, TN, pH, and TK; Figure 1), suggesting these variables are closely linked within the managed soil ecosystem. Furthermore, the trends of change for OM, TN, and pH exhibited distinct regional patterns. This indicates that the long-term application of P fertilizers at different weights has not only created distinct soil P levels but has also jointly altered soil chemical properties, with the nature and magnitude of these co-variations being highly site-specific. The similar trends between OM, TN and P levels at the SH site suggests that P fertilization may have directly or indirectly enhanced primary productivity and subsequent organic matter return to the soil (Shi et al., 2022). Conversely, the inverse trend observed at the YD site could be attributed to a more complex interaction. At YD, the historically higher background fertility and potentially greater reliance on organic amendments might have established a different nutrient balance. Here, elevated P input might increase soil microbial biomass and respiration (Spohn and Schleuss, 2019), which accelerated the decomposition of SOM (Coonan et al., 2019; Kirkby et al., 2014), leading to unexpected shifts in OM and TN pools. In the SH site, high P input led to a significant increase in soil pH (Figure 1). This increase can be attributed to the strong specific adsorption of phosphate ions by iron and aluminum oxides in this acidic soil, a process that consumes H^+^ from the soil solution and releases OH^−^, thereby raising the pH (Barrow, 2017; Zhu et al., 2018). Notably, the magnitude of this effect depends on the content of soil oxides and the initial pH (Li et al., 2016), this helps explain why the same trend was not observed in the other regions (YD and ZS), likely due to differences in their soil mineral composition or initial chemical properties. The lack of significant changes in OM, TN, and pH across P levels at ZS likely reflects its unique soil constraints (e.g., higher TK and SRK, sandy soil type), which collectively may have buffered the soil system against the chemical perturbations induced by differential P input.

Soil phosphatases catalyze the mineralization of organic P, converting organic P into plant-available forms, and thus serve as a bridge connecting the soil organic P pool and the plant-available P pool (Wu et al., 2026). Previous studies have suggested a negative feedback regulation between soil phosphatase activity and available P content (Margalef et al., 2021). When soil AP is deficient, plants and microorganisms perceive P stress to acquire essential P, they actively secrete more phosphatases into the soil, thereby alleviating the P stress (Ragot et al., 2017; Samaddar et al., 2019). However, this study found a highly significant positive correlation between soil phytase activity and AP content (Figure 2). This phenomenon aligns with observations from a previous study by Zhou et al. (2024) and may indicate that phytase activity in P-rich soils is no longer driven by P deficiency signals. This pattern could be jointly driven by the following mechanisms. First, higher P levels, co-varying with increased OM (Figure 1), likely support a larger and more active overall microbial biomass (Jiang et al., 2025; Paliaga et al., 2025). Thus, the observed higher total enzyme activity may reflect a greater pool of enzyme producers rather than a per-cell increase in synthesis. Second, long-term P fertilization have selected for functionally specialized microbial communities adapted to higher P availability (Leff et al., 2015). These communities might maintain a constitutively high expression of phosphatase to rapidly mineralize organic P inputs. This aligns with functional prediction showing site-specific enrichment of P-cycling genes (Figure 6). Finally, the positive correlation could stem from a feedback loop where higher AP supports greater plant growth and root exudation, providing more organic substrates that, in turn, stimulate microbial activity and enzyme production. Our observation that enriched ASVs correlated with both AP and phytase activity (Supplementary Figure S2) supports the existence of such a microbial group contributing to both P availability and organic P mineralization. Similar positive correlations in managed systems have been reported, emphasizing the context-dependency of microbial nutrient acquisition strategies (Estrela et al., 2021). Therefore, in this ecosystem, phytase activity may primarily reflect the microbial community's response to the availability of organic P substrates and its own metabolic demands, rather than a simple stress response to inorganic P scarcity. This finding underscores the profound influence of agricultural management practices in reshaping the functional strategies of soil microbial communities.

### 4.2 Site-specific environment overrides phosphorus level in shaping community structure

Despite significant variations in P levels, bacterial alpha diversity and the relative abundance of major phyla showed minimal response to P gradients within each site (Figure 3, Supplementary Figure S1). Instead, bacterial community structure exhibited fundamental differentiation among the three sites (YD, SH, ZS), while the differences among soils with different P levels within the same site were far less pronounced than the inter-site differences (Figure 4). The heterogeneous dispersion at the SH site suggests that P levels may alter the strength of ecological filtering or stochastic processes, leading to divergent within-group variability. In contrast, the homogeneous dispersion at YD and ZS implies consistent assembly processes across P gradients.

Higher soil P levels led to the enrichment of certain bacterial ASVs and a concomitant decrease in the abundance of others (Figure 5). β-diversity partitioning analysis further confirmed that the divergence of bacterial communities across different P levels was primarily driven by species replacement (Supplementary Figure S5). These results collectively underscore the preeminent role of geographical location and its associated environmental factors (e.g., parent material, long-term management practices, climatic conditions, and local soil properties) over contemporary P fertilization regimes in shaping the foundational composition of soil bacterial communities (Hartmann and Six, 2023). The strong site-specific of the enriched bacterial ASVs (Figure 5) indicates that the local species pool and environmental filters are dominant forces in community assembly, a finding consistent with the concept of “everything is everywhere, but the environment selects” in microbial biogeography (de Wit and Bouvier, 2006).

Different levels of P fertilizer significantly influence the assembly process of crop rhizosphere microbial communities, due to a reduction in the abundance of oligotrophic bacteria (from Acidobacteria and Chloroflexi) within key microbial groups, the community assembly process shifts from homogeneous dispersal to variable selection, and is significantly regulated by soil factors (total P and carbon-to-nitrogen ratio; Wu et al., 2023). While in this study, P level acts as a secondary, yet significant, deterministic filter among these three sites. It drives species replacement (evidenced by different enriched ASVs across P levels, Figure 5) rather than causing nested subsets, fine-tuning the community without overhauling its core structure. In conclusion, the assembly of bacterial communities in these tobacco soils may be governed by a hierarchical filtering process: first, a strong regional filter determined by site-specific factors could establish distinct foundational communities; second, within each region, P availability might act as a local environmental filter, primarily driving species replacement and functional fine-tuning rather than wholesale community restructuring. This proposed hierarchical assembly process may help explain why functional potentials (Figure 6) were more distinct between sites than within them. This perspective offers a plausible way to reconcile the seemingly minor within-site structural changes with the significant functional correlations (e.g., phytase activity with AP). A potential mechanism is that the key microbial groups mediating these functions could be among the taxa that are replaced, with their relative abundances shifting in response to P levels without overhauling the entire community structure.

The distinct co-occurrence network topology observed in high-AP soils, characterized by increased overall connectivity coupled with decreased local clustering (Figure 7), may reflect a shift in the ecological forces structuring the microbial community. In low-AP soils, environmental filtering and niche competition might favor the development of specialized, tightly-knit modules (high clustering), where taxa interact intensely within small groups to exploit scarce resources. The alleviation of P limitation in high-AP soils could relax these constraints, leading to non-exclusive outcomes. This pattern is consistent with the concept that nutrient enrichment can simplify interaction networks by favoring habitat generalists over niche specialists (Hernandez et al., 2023; Yuan et al., 2026). While our correlative data cannot definitively distinguish mechanisms, the observed network architecture provides evidence that long-term phosphorus management does not merely change the composition of the microbial community, but may also fundamentally alter the blueprint of microbial interactions, with potential implications for community stability and functional resilience.

### 4.3 Site-specific functional repertoires and their environmental drivers

Distinct P metabolism gene profiles were observed across the YD, SH, and ZS sites (Figure 6), reflecting divergent microbial strategies for P cycling shaped by local conditions. Results *via* PICRUSt2 suggest that the YD community is functionally geared toward aggressive P acquisition and release. In contrast, the SH and ZS communities exhibited a higher genetic potential for polyphosphate degradation and transport, indicating a strategy oriented toward efficient P scavenging, intracellular storage, and recycling, a profile that may be adaptive in environments with stronger P competition or fluctuation. The elevated abundance of the predicted regulatory genes at the ZS site corresponds to its lower total P content, suggesting a microbial community under tighter metabolic control to maintain P homeostasis, a response consistent with findings in other nutrient-limited soils (Bergkemper et al., 2016). These functional variations underscore how soil properties such as pH, organic carbon, and oxygen availability shape microbiome composition and function (Philippot et al., 2023). Previous studies have similarly shown that shifts in soil P availability can significantly alter bacterial community structure and functional traits, often increasing the abundance of P-cycle-related enzymes under P-deficient conditions (Samaddar et al., 2019).

The functional differences observed are closely associated with site-specific environmental drivers, as revealed by RDA analysis (Figure 8). At the YD site, OM was the primary factor shaping the bacterial community. In contrast, the community at the SH site was influenced by a combination of OM and multiple nutrients (TK, TN, TP, and AK), reflecting a more complex, multi-resource interactive environment. Notably, at the ZS site, potassium (TK, AK) emerged as the dominant driver. This clear shift in key environmental filters, from OM to potassium, across a small geographic scale strongly demonstrates how localized soil properties determine microbial ecological niches and functional potential. The prominent roles of OM and nutrient conditions indicate that managing soil chemistry, especially through organic amendments, can actively influence microbially mediated P cycling. This is supported by studies showing that organic agricultural management enhances soil P availability primarily by increasing the potential for inorganic P solubilization, whereas inorganic management tends to regulate P availability by altering the potential for organic P mineralization (Liao et al., 2023). For example, partially replacing chemical P fertilizer with manure or crop straw has been shown to improve P-use efficiency. This effect is attributed to the high-molecular-weight dissolved organic matter derived from these amendments, which competes with P for sorption sites and reduces soil surface charge, thereby inhibiting P fixation in systems such as paddy soils (Wang et al., 2024). Furthermore, organic amendments are known to stimulate microbial P cycling by enriching related functional genes. Specifically, the abundance, rather than the diversity, of key genes such as *phoD* or *phoC* has been identified as a major regulator of soil alkaline or acid phosphatase activity (Wu et al., 2025). These findings align with broader evidence that agricultural management practices (e.g., tillage frequency, herbicide application) and inherent soil properties (e.g., pH) are key determinants of P-cycling microbial community structure (Long et al., 2026). Collectively, this evidence underscores that management strategies tailored to local soil conditions can effectively modulate the microbial functional processes governing P availability.

### 4.4 Functional fine-tuning *via* enriched taxa within sites

Notably, with the exception of polyphosphate synthesis at the ZS site, no significant differences were observed in the total abundance of P-cycling functional genes among soils with different P levels within the same site. This indicates that, at the scale of this study, differences in soil available P content did not lead to fundamental changes in the genetic functional structure of P-metabolizing microbial communities. Instead, microbial communities likely respond to variations in P availability primarily by regulating gene expression levels (e.g., upregulating phytase activity) rather than through extensive enrichment of relevant functional groups. This functional resilience within sites contrasts sharply with the strong functional divergence between sites, underscoring that geographic legacy combined with the influence of local soil properties, crop genotype, and management history set the core functional template, which is then dynamically fine-tuned at the transcriptional level in response to current P availability.

The ASVs enriched in higher-P soils and correlated with both AP and phytase activity provide clues about the taxa mediating this fine-tuning and the observed P dynamics (Supplementary Figure S2). For instance, *Pseudomonas* genera (enriched in YD) were known as phosphate-solubilizing bacteria (Peng et al., 2025), *Gaiellales* order (enriched in SH) actinomycetes also have clear P solubilization potential (Hamdali et al., 2008). Their co-occurrence with high AP and phytase activity suggests they may be key actors in a potential positive feedback loop that maintains high P availability. Conversely, the ASVs that decreased in abundance may represent oligotrophic specialists outcompeted in a more resource-rich environment. This functional replacement aligns with the observation that P metabolic potentials varied significantly between regions but were less responsive to P levels within a region (Figure 6), which may imply that location selects for the functional potential, while local P levels fine-tune its expression through shifts in specific taxa.

### 4.5 Synthesis and implications

Our study demonstrates that in these acidic tobacco soils, the microbial mediation of the P cycle is decoupled from simple resource scarcity principles. Instead, it may be governed by a hierarchy of controls: (1) Site-specific environment determines the foundational species pool and major functional potential; (2) Local soil chemistry (OM, K, etc.), often a product of management history, acts as the primary environmental filter shaping both community structure and functional gene repertoire; and (3) Contemporary P level fine-tunes the community through species replacement and likely regulates the expression of existing functional genes (as suggested by the phosphatase activity response), rather than altering the core functional genetic capacity.

From an agronomic perspective, this implies that soil management practices aimed at enhancing P availability in SH, YD, and ZS must consider the local soil context and its established microbial functional profile. A one-size-fits-all approach is unlikely to succeed. For instance, in a soil like YD's, managing OM to sustain the native P-solubilizing/mineralizing community might be key. In contrast, in ZS-like soils, managing potassium balance and fostering a community efficient in P storage and recycling could be more important. It is important to note that the genetic potentials observed by PICRUSt2 predictions need validation *via* metagenomics, metatranscriptomics, or enzyme kinetics. In addition, this observational, cross-sectional study does not allow for the full separation of historical management legacies from contemporary soil chemistry effects. Instead, it establishes a foundational ecological framework linking soil P gradients to bacterial community properties, which can guide future mechanistic hypotheses and targeted experimental work. Future strategies could move beyond managing just P inputs toward managing the soil habitat to cultivate a microbial community capable of maintaining efficient P cycling under local conditions.

## 5 Conclusions

This study systematically reveals the impact of long-term P application on the acidic tobacco-growing soil properties and bacterial communities. The research found a significant positive correlation between soil phytase activity and available P content. This functional response exhibits high spatial heterogeneity: bacterial community structure, diversity, and the predicted P cycling functional gene profiles are primarily governed by site-specific environment, while differences in P levels fine-tune the community within this established framework by driving species replacement. RDA further linked the predicted functional differentiation among sites (YD dominated by mineralization/solubilization, SH/ZS by turnover/storage) to key environmental drivers (YD driven by OM, SH by multi-factor synergy, and ZS dominated by potassium), demonstrating the decisive role of environmental selection at the local scale. In summary, the distinct bacterial community structure and functional potential were primarily shaped by the composite, site-specific context (encompassing local soil type, management history, and cultivar), rather than by the contemporary gradient in soil P levels, current P management performs active fine-tuning. Therefore, management strategies to enhance soil P efficiency should shift toward precise ecological regulation, that is, cultivating adapted soil microbial functional communities based on locally dominant environmental drivers, such as organic matter or potassium balance.

## Data Availability

The datasets presented in this study can be found in online repositories. The names of the repository/repositories and accession number(s) can be found below: https://www.ncbi.nlm.nih.gov/, PRJNA1460904.
