# Supplementary material for: Microbial community assembly and functional potential response driven by soil phosphorus gradients
Source: Front Microbiol. 2026 Jun 26;17:1818929. doi: 10.3389/fmicb.2026.1818929 (PMC13350453; doi:10.3389/fmicb.2026.1818929)
Supplement: Supplementary file 1 [file Table_1.DOCX]

# Figure S1


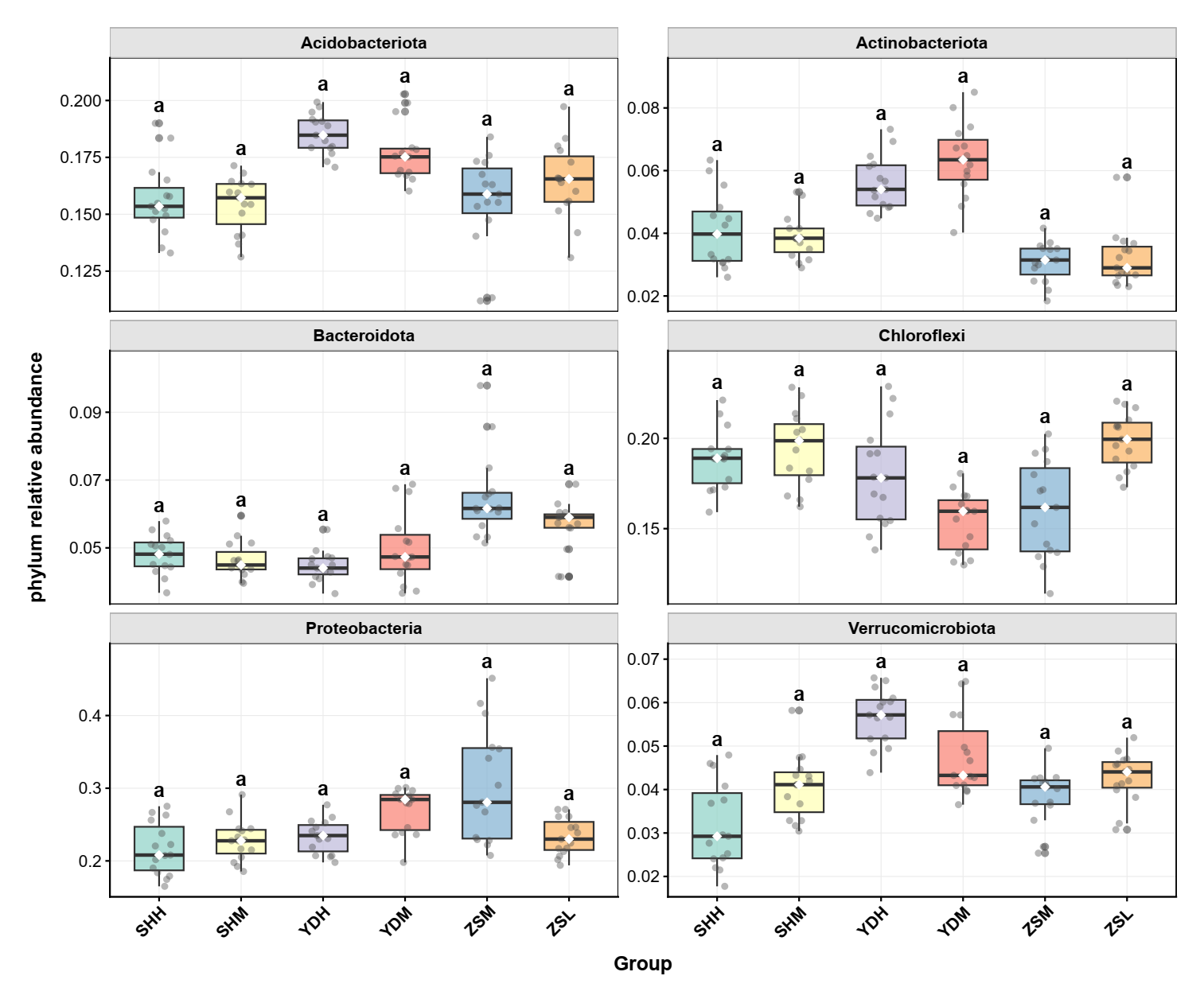


Figure S1 The relative abundances of the main bacterial phylum in soils with different phosphorus levels across three sites. YD: Yongding, SH: Shanghang; ZS: Zhongsha. The letters H, M, and L following the site abbreviations denote high, middle, and low levels of soil available phosphorus (AP), respectively.

# Figure S2


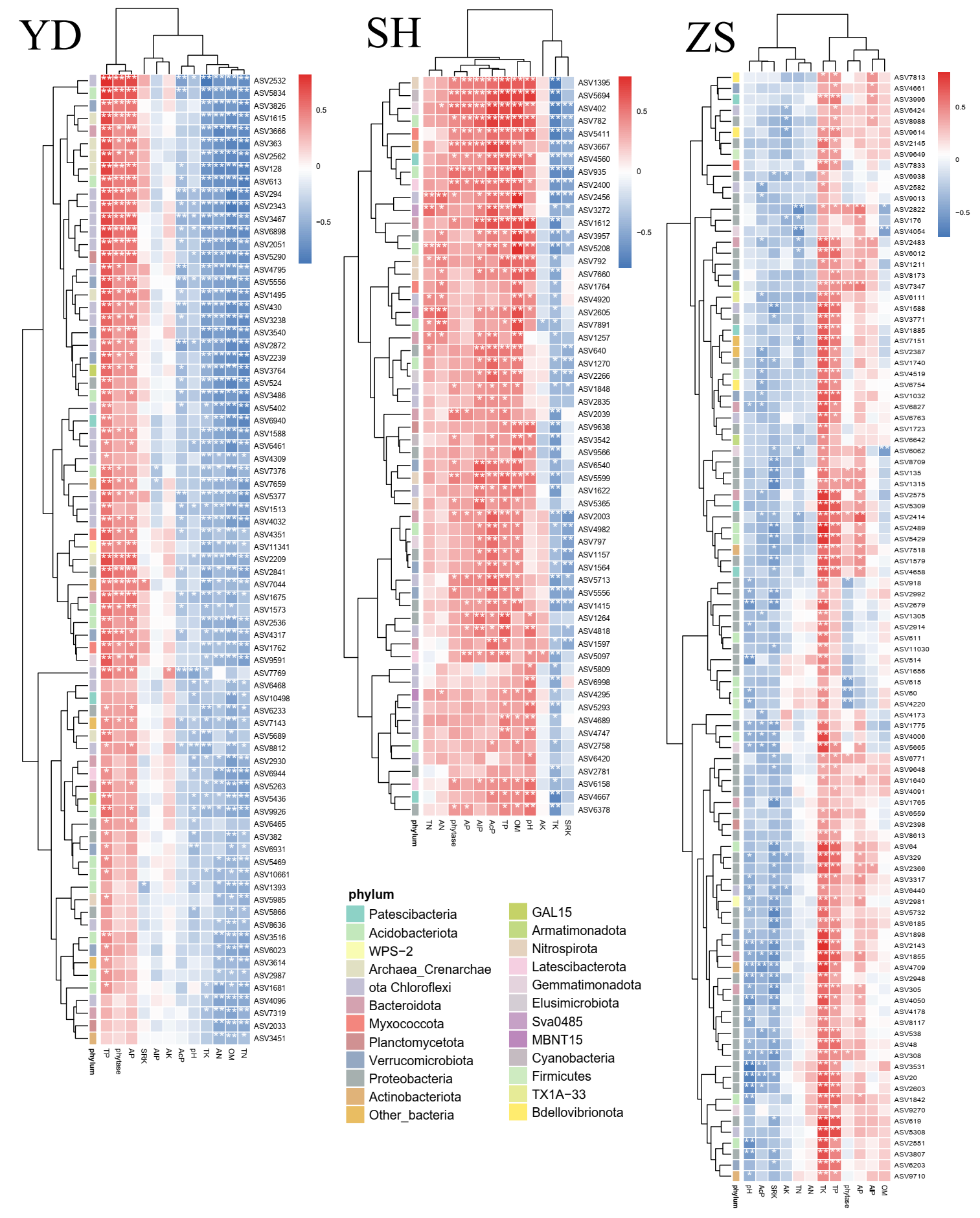


Figure S2. Heatmap of spearman correlations between enriched ASVs and soil chemical properties or enzyme activity. Colors represent Spearman's rank correlation coefficients. Asterisks denote statistically significant correlations after FDR correction: *, FDR-adjusted *p*< 0.05; **, *p* < 0.01. YD: Yongding, SH: Shanghang; ZS: Zhongsha.

**Figure S3**


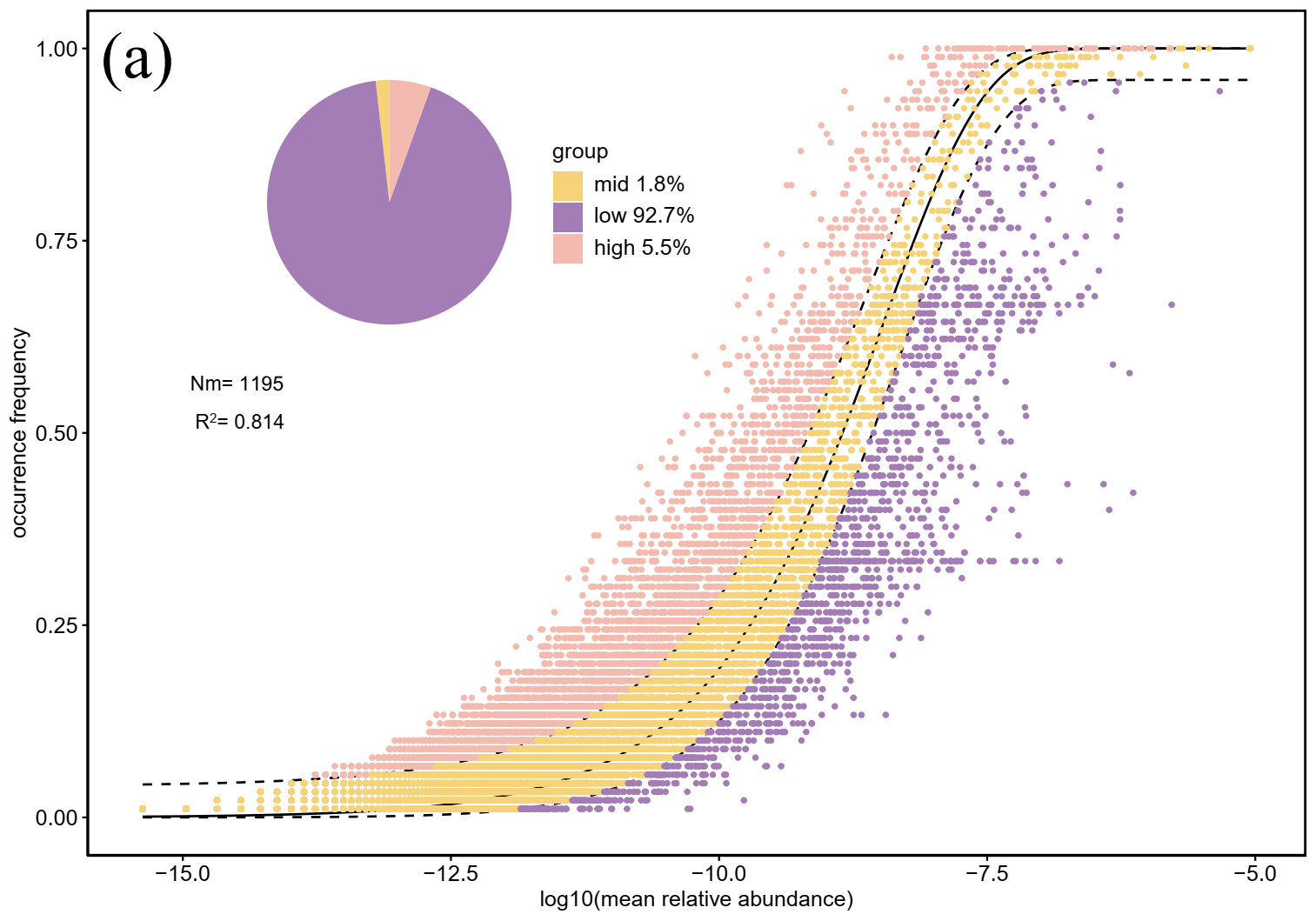


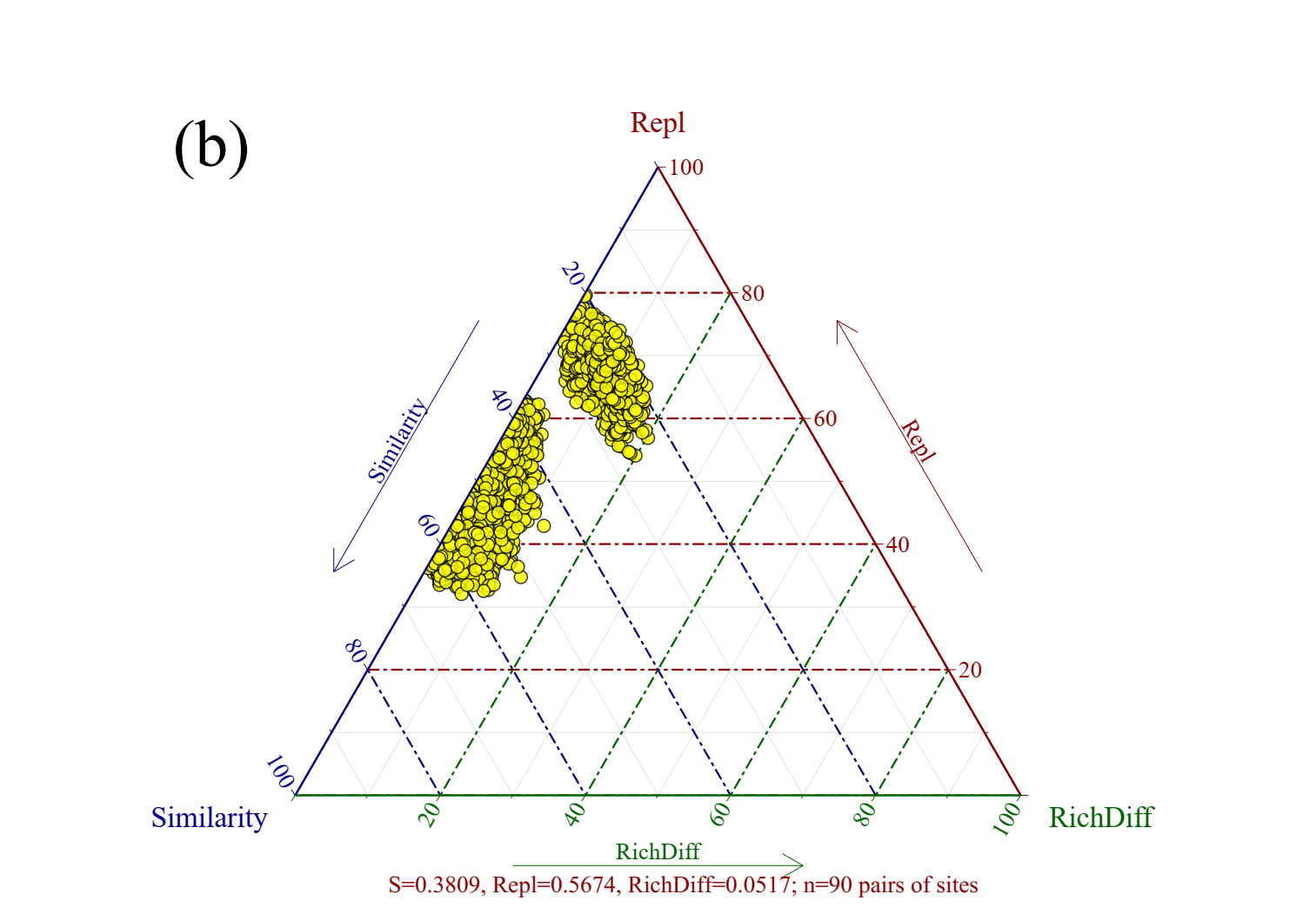


Figure S3. (a) Fit of the Neutral community model of bacterial community assemblage in soils with different phosphorus levels across three sites. The solid black lines indicate the best fit to the NCM, and the dashed red lines represent 95% confidence intervals around the model prediction. Genera that occur more or less frequently than those predicted by the NCM are shown in different colors. R^2^ indicates the fit of the model. (b) Triangular plots (simplices) show community ecological processes (i.e., species replacement and richness difference) of bacteria among the 8010 pairs of samples. Each yellow point represents a pair of samples. Its position is denoted by a triplet of values from the Similarity (S) = 1 – D (dissimilarity), Repl (replacement), RichDiff (richness difference) matrices; each triplet sums to 1.

# Figure S4


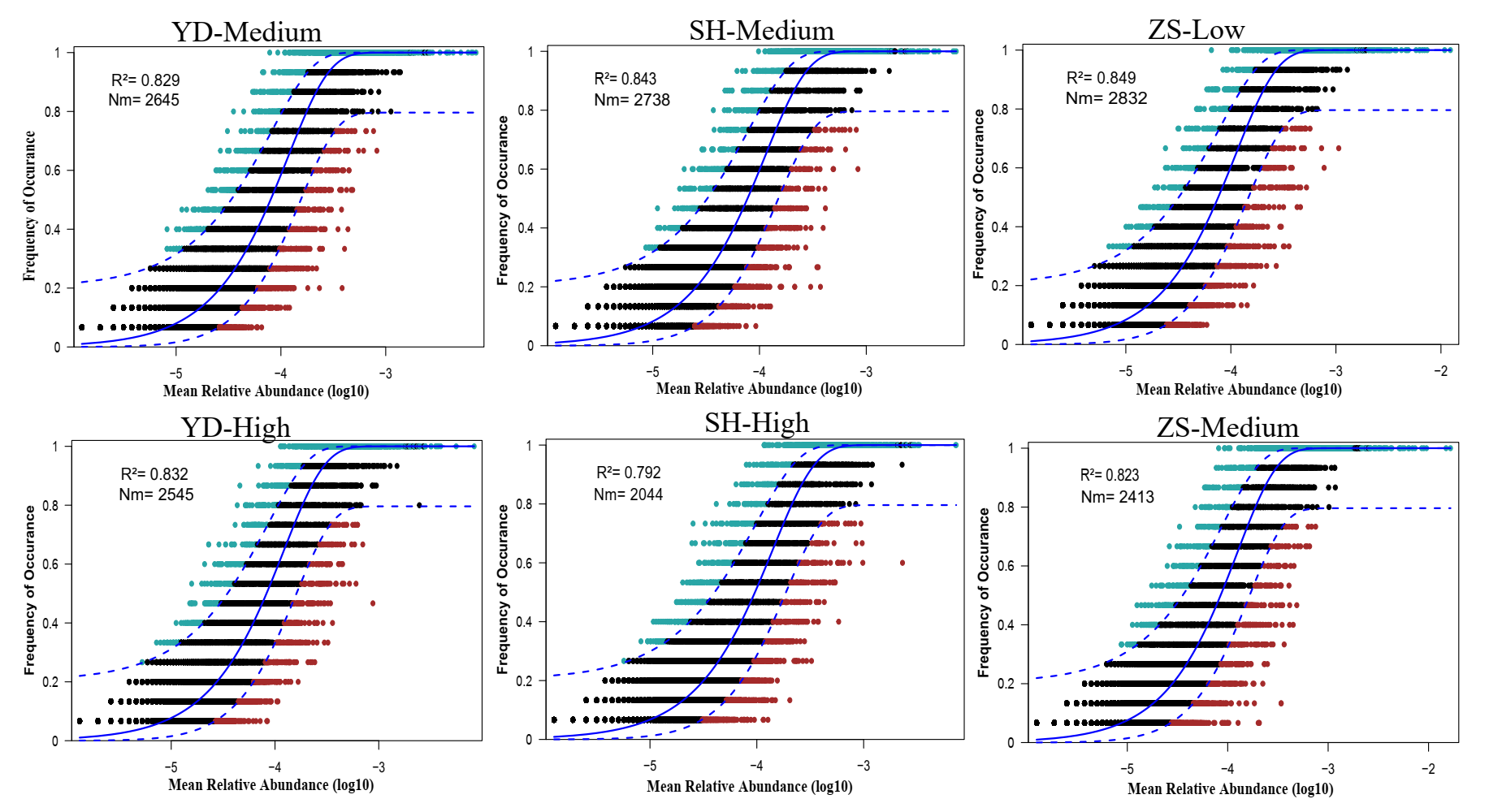


Figure S4. Fit of the Neutral community model of microbial community assemblage in soils with different phosphorus levels across three sites. The solid black lines indicate the best fit to the NCM, and the dashed red lines represent 95% confidence intervals around the model prediction. Genera that occur more or less frequently than those predicted by the NCM are shown in different colors. R2 indicates the fit of the model.

# Figure S5


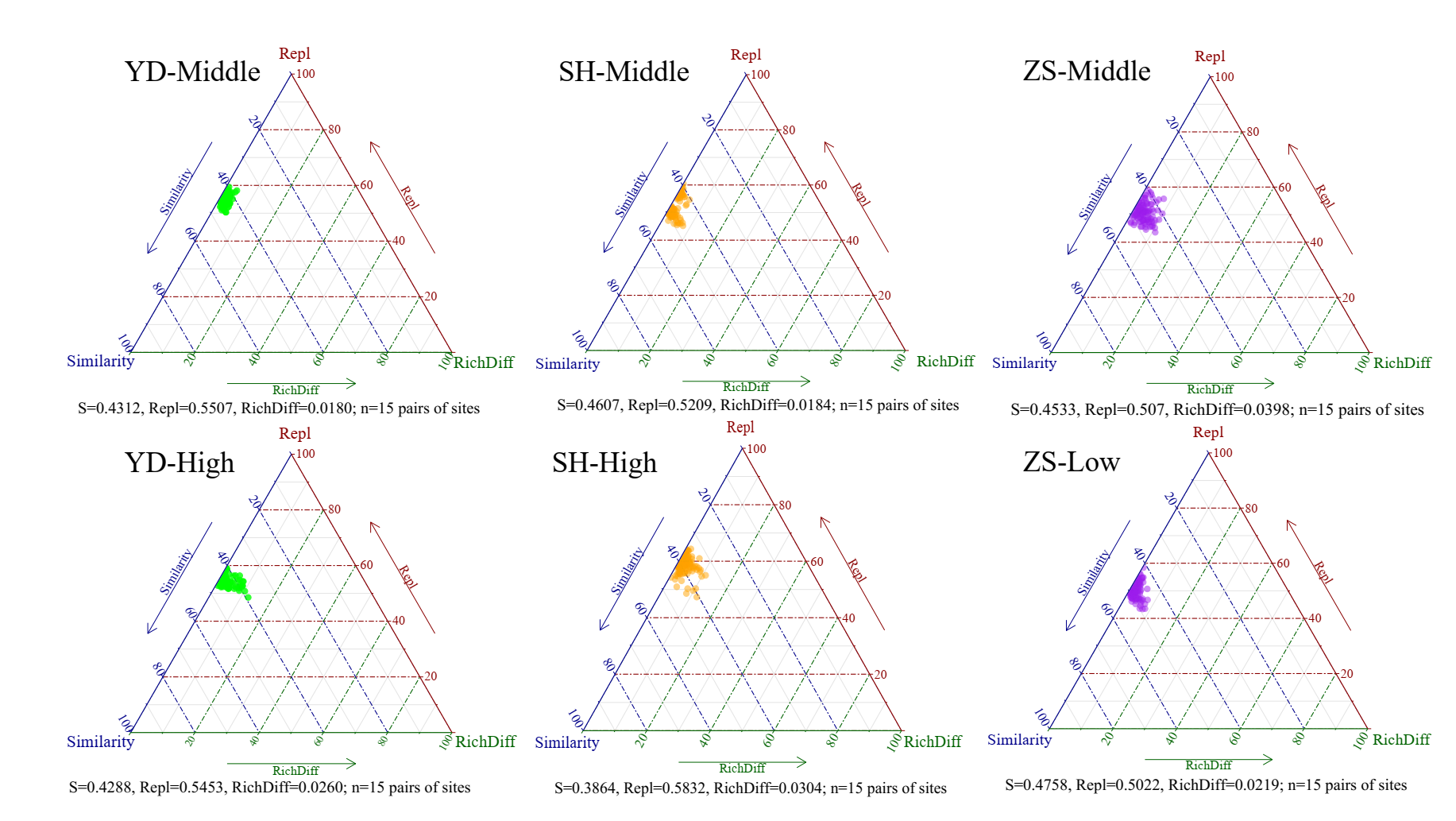


Figure S5. Triangular plots (simplices) show community ecological processes (i.e., species replacement and richness difference) of bacteria among the 1335 pairs of samples. Each point (blue, orange or purple dots) represents a pair of samples. Its position is denoted by a triplet of values from the Similarity (S) = 1 – D (dissimilarity), Repl (replacement), RichDiff (richness difference) matrices; each triplet sums to 1.

# Table S1

Information of functional genes involved in the P cycling processes identified in this study.

| KEGG_Description | KO_number | gene | KO_Description |
| --- | --- | --- | --- |
| Organic P mineralization | K01077 | phoA | alkaline phosphatase [EC:3.1.3.1] |
| Organic P mineralization | K01113 | phoD | phosphodiesterase/alkaline phosphatase D [EC:3.1.4.1];alkaline phosphatase D [EC:3.1.3.1] |
| Organic P mineralization | K03430 | phnW | 2.6.1.37 |
| Organic P mineralization | K05306 | phnX | 3.11.1.1 |
| Organic P mineralization | K05774 | phnN | 2.7.4.23 |
| Organic P mineralization | K05780 | phnL | 2.7.8.37 |
| Organic P mineralization | K06162 | phnM | 3.6.1.63 |
| Organic P mineralization | K06163 | phnJ | 4.7.1.1 |
| Organic P mineralization | K06164 | phnI | 2.7.8.37 |
| Organic P mineralization | K06165 | phnH | 2.7.8.37 |
| Organic P mineralization | K06166 | phnG | 2.7.8.37 |
| Organic P mineralization | K06167 | phnP | 3.1.4.55 |
| Organic P mineralization | K09474 | phoN | 3.1.3.2 |
| Organic P mineralization | K19670 | phnA | 3.11.1.2 |
| Organic P mineralization | K01093 | appA | 4-phytase / acid phosphatase [EC:3.1.3.26 3.1.3.2] |
| Organic P mineralization | K01083 |  | 3-phytase [EC:3.1.3.8] |
| Organic P mineralization | K01078 | olpA | acid phosphatase [EC:3.1.3.2] |
| Organic P mineralization | K03788 |  | acid phosphatase (class B) [EC:3.1.3.2] |
| Inorganic P solubilization | K00117 | gcd | 1.1.5.2 |
| Inorganic P solubilization | K01507 | ppa | 3.6.1.1 |
| Inorganic P solubilization | K01524 | ppx-gppA | 3.6.1.11; 3.6.1.40 |
| Regulatory | K02039 | phoU | phosphate transport system protein |
| Regulatory | K07636 | phoR | 2.7.13.3 |
| Regulatory | K07657 | phoB | two-component system, OmpR family, phosphate regulon response regulator PhoB |
| Regulatory | K07660 | phoP | two-component system, OmpR family, response regulator PhoP |
| Transporters | K01126 | glpQ | 3.1.4.46 |
| Transporters | K02036 | pstB | 7.3.2.1 |
| Transporters | K02037 | pstC | phosphate transport system permease protein |
| Transporters | K02038 | pstA | phosphate transport system permease protein |
| Transporters | K02040 | pstS | phosphate transport system substrate-binding protein |
| Transporters | K02041 | phnC | 7.3.2.2 |
| Transporters | K02042 | phnE | phosphonate transport system permease protein |
| Transporters | K02043 | phnF | GntR family transcriptional regulator, phosphonate transport system regulatory protein |
| Transporters | K02044 | phnD | phosphonate transport system substrate-binding protein |
| Transporters | K03306 | TC.PIT | inorganic phosphate transporter, PiT family |
| Transporters | K05781 | phnK | putative phosphonate transport system ATP-binding protein |
| Transporters | K05813 | ugpB | sn-glycerol 3-phosphate transport system substrate-binding protein |
| Transporters | K05814 | ugpA | sn-glycerol 3-phosphate transport system permease protein |
| Transporters | K05815 | ugpE | sn-glycerol 3-phosphate transport system permease protein |
| Transporters | K05816 | ugpC | 7.6.2.10 |
| Polyphosphate synthesis | K00937 | ppk1 | 2.7.4.1 |
| Polyphosphate synthesis | K15986 | ppaC | 3.6.1.1 |
| Polyphosphate degradation | K00858 | ppnK | 2.7.1.23 |
| Polyphosphate degradation | K00873 | PK | 2.7.1.40 |
| Polyphosphate degradation | K00886 | ppgK | 2.7.1.63 |
| Polyphosphate degradation | K00940 | ndk | 2.7.4.6 |
| Polyphosphate degradation | K00951 | relA | 2.7.6.5 |
| Polyphosphate degradation | K01139 | spoT | 2.7.6.5; 3.1.7.2 |
| Polyphosphate degradation | K03787 | surE | 3.1.3.5; 3.1.3.6 |
| Polyphosphate degradation | K21138 | HDDC3 | 3.1.7.2 |
| Polyphosphate degradation | K22468 | ppk2 | 2.7.4.34 |
| Polyphosphate degradation | K23753 | pap | 2.7.4.33 |

# Table S2

Table S2 Network topological properties of bacterial communities in soils with different available phosphorus (AP) levels across three sites

| Network indices | YDM | YDH | SHM | SHH | ZSL | ZSM |
| --- | --- | --- | --- | --- | --- | --- |
| Node | 2297 | 2249 | 1828 | 3538 | 1547 | 2494 |
| Edge | 2891 | 2535 | 2032 | 8739 | 1539 | 4927 |
| Negative correlated edges | 66 | 89 | 58 | 912 | 36 | 193 |
| Positive correlated edges | 2825 | 2446 | 1974 | 7827 | 1503 | 4734 |
| Network clustering coefficient | 0.700 | 0.672 | 0.75 | 0.432 | 0.629 | 0.605 |
| The average path length | 5.94 | 3.481 | 3.06 | 8.811 | 1.961 | 9.424 |
| Network density | 0.0011 | 0.0010 | 0.0012 | 0.0014 | 0.0013 | 0.0015 |
